# Pathogenetic and Therapeutic Applications of Tumor Necrosis Factor-α (TNF-α) in Major Depressive Disorder: A Systematic Review

**DOI:** 10.3390/ijms17050733

**Published:** 2016-05-14

**Authors:** Ke Ma, Hongxiu Zhang, Zulqarnain Baloch

**Affiliations:** 1 Department of Physiology, Medical College of Qingdao University, Qingdao 266071, China; 2 Faculty of Life Science and Technology, Kunming University of Science and Technology, Kunming 650500, China; xiaoxiu1009@163.com

**Keywords:** major depressive disorder, tumor necrosis factor-α, pathophysiology, genetic polymorphism, therapeutic

## Abstract

Major depressive disorder (MDD) is characterized by mood, vegetative, cognitive, and even psychotic symptoms and signs that can cause substantial impairments in quality of life and functioning. Up to now, the exact pathogenesis of MDD remains poorly understood. Recent research has begun to reveal that the pro-inflammatory cytokines, particularly, tumor necrosis factor-α (TNF-α), play an integral role in the pathophysiology of depressive disorders and the mechanism of antidepressant treatment. On the base of several observations: it is found that subsets of MDD patients have enhanced plasma levels TNF-α; antidepressant treatments had linked with the decline of TNF-α; central administration of TNF-α gives rise to sickness behavior which shares features with depression; and a blockade of it can ameliorate depressive symptomatology in animal models and clinical trials. In this review article, we focus on recent evidence linking TNF-α and MDD looking at data from animal and clinical studies, illustrating the pathophysiological role, susceptibility and its therapeutic application in depression. We conclude by discussing future directions for research, in particular the opportunities for the development of novel therapeutics that target TNF-α. This will be very important for designing preventative strategies and for the identification of new drug targets and preventative strategies.

## 1. Introduction

Major depressive disorder (MDD) is a widespread, diverse and intermittent neuropsychiatric disorder characterized by a wide range of symptoms, such as cognitive functions and altered mood [1,2,3]. Almost 50% of depression patients express suicidal thoughts and tendencies at some stage in their lives; among them, 10%–15% ultimately pledge suicide [4]. Interestingly, about 50% patients do not recover following an antidepressant treatment, while 20% of them fail to answer to any intervention [5]. It has been predicted that depression will become the second-leading cause of disability worldwide after human immunodeficiency virus (HIV) in 2030 [6]. Extensive research efforts have been done in past decades; however, the etiology of depression is still indefinable, its diagnosis unclear and the pharmacotherapy ineffective. This may be due to poor understanding of the molecular pathophysiology of depression.

It has been widely accepted that depression may be a collection of partly distinct diseases, with overlapping causal pathways, initiating from the interaction among environmental and genetic factors [7,8]. Alterations in several interacting systems are likely to cause depression. Thus, several hypotheses have been suggested to explain its origins. Inflammatory hypothesis is one of them, which was initially proposed by Smith called the “macrophage” theory of depression, and currently also called the “malaise or cytokine” theory of depression [9]. This hypothesis underlines the importance psycho-neuroimmunological dysfunction where there is stimulation of the immune system. Certainly, MDD patients have an abnormal peripheral immune system [10], with weak cellular immunity, and high levels of pro-inflammatory cytokines such as tumor necrosis factor (TNF)-α, interferon (IFN)-γ, and interleukin (IL)-1β, IL-2, IL-6, IL-8 [11]. Additionally, it has been shown that pro-inflammatory cytokines have an effect on pathophysiological domains, such as neuroendocrine function, regional brain activity and neurotransmitter metabolism, all of these may contribute to the pathogenesis of MDD [12]. Moreover, data collected from some preclinical studies and animal models have also supported the causal nature of this theory, in which injecting high levels of pro-inflammatory cytokines cause depression-like symptoms and behavior and also provide evidence that patients with inflammation-based treatments are more inclined to suffer depressed mood or depressive disorder [13,14,15].

Among the pro-inflammatory cytokines, multiple studies have focused on TNF-α being an endogenous pyrogen, which can cause inflammation, apoptotic cell death, and mediate the release of variety cytokines like IL-6, IL-8 and IL-1β by stimulated macrophages [16,17]. Dysregulation, especially overproduction of TNF-α, has been found in a variety of human diseases including atherosclerosis [18], cancer [19], atherosclerosis [20] and inflammatory bowel disease [21]. However, it is still unclear, and many studies of depression are currently being connected to the TNF-α level. Recent meta-analyses have demonstrated very high concentrations of TNF-α, IL-6 and IL-1β in depressed patients as compared with healthy controls [22,23]. However, currently reported genome-wide association study (GWAS) about MDD only found that TNF-α was genetically associated with MDD among proinflammatory cytokines [24]. Furthermore, in animal studies, TNF-α parental root administration caused depressive-like symptoms in rodents [25]. While the molecular mechanisms of TNF-α in regulating MDD are unclear, accumulated evidence from clinical studies found that a reduction of TNF-α in the blood level was linked with progress in depressive symptoms, and effective treatment of MDD with antidepressants or electroconvulsive therapy normalized the blood level of TNF-α [26]. Additionally, a blockade of TNF-α by its pharmacological inhibitors can inverse the depressive symptoms of patients [27,28].

Taken together, this evidence suggested that, compared to other inflammatory cytokines, TNF-α may play an integral part in the progress of MDD and the mechanism of antidepressant treatments. Previous studies have simply reviewed the experimental and cross-sectional studies that have supported the association of elevated serum concentrations of pro-inflammatory cytokines in depression. However, articles rarely aim to comprehensively review the leading role of specific inflammatory cytokines in depression from aspects of pathophysiological, genetic susceptibility, therapeutic applications and so on. Therefore, we integrate recent progress of the relationship between TNF-α and depressive disorders. In this paper, we begin review by providing a brief overview of TNF-α and the central nervous system (CNS), and then summarize evidence from clinical and animal studies, which suggest that TNF-α may be capable of causing mood swings and depression. Then, we focus on the more extensive rodent and human primate literature to illustrate the pathogenesis, susceptibility and therapeutic role of TNF-α in depression. We further elaborate on the role of TNF-α in depression with autoimmune diseases. We conclude by discussing future directions for research, in particular, the opportunities for the development of novel therapeutics that target TNF-α.

## 2. Tumor Necrosis Factor (TNF)-α and the Central Nervous System (CNS)

### 2.1. TNF-α and Signaling Pathways

TNF-α is produced from various kinds of cells such as lymphoid cells, cardiac myocytes, activated macrophages, endothelial cells, mast cells, fibroblasts, neurons and adipose tissue [29]. It is primarily secreted as a 212-amino acid-long type II trans-membrane protein arranged in stable homotrimers [30]. From this membrane, a combined form the soluble homotrimeric cytokine (sTNF) is released through proteolytic cleavage by the metalloprotease TNF-α converting enzyme (TACE). The soluble 51 kDa trimeric sTNF commonly detaches at concentrations less than the nanomolar range, thus losing its bioactivity. The secreted form of human TNF-α develops a triangular pyramid shape, and weighs around 17-kDa. Although the definite function of them is still controversial, both forms may have overlapping and distinct biology activities [31].

TNF-α has multi-biological effects primarily by binding to tumor necrosis factor receptors 1 (p55 receptor) and tumor necrosis factor receptors 2 (p75 receptor), giving rise to stimulation of complex signaling cascades that control various intracellular functions [32]. TNFR1 has been expressed in many tissues and can be completely motivated by both the soluble trimeric and membrane-bound forms of TNF-α, while TNFR2 is restricted to the cells of the immune system and can only react to the membrane-bound form of the TNF homotrimer [33]. Maximum data about the TNF signaling pathway have been derived from TNFR1, while the role of TNFR2 remains unclear. TNFR is comprised of several members with homologous cytoplasmic domains called as death domains (DD). The intracellular DD are vital for starting apoptosis and other signaling pathways after ligand binding with the receptors [34]. When TNF-α bind with their ligand, it triggers a conformational change in the receptor, resulting in the detachment of the inhibitory protein silencer of death domains (SODD) from the intracellular death domain. This dissociation enables the adaptor protein, a TNFR1 related death domain protein (TRADD) to attach with the death domain, acting as a basis for subsequent protein binding, which activates the following three pathways: activation of the NF-κB pathway, stimulation of the mitogen-activated protein kinase (MAPK) pathway and stimulation of the death signaling pathway [35,36]. The myriad and contradictory effects are controlled through the above-mentioned pathways show the presence of extensive cross-talk. As an example, NF-κB increases the transcription of inhibitory proteins, which interfere with death signaling, while activated caspases cut some components of the NF-κB pathway. This complex signaling confirms that, when TNF is released, various cells with very dissimilar roles and circumstances can all respond appropriately to inflammation [37].

### 2.2. Production of TNF-α within CNS

It is well established that CNS is an immune-privileged organ. However, immune status is not clear and fluctuates with age and brain region [38]. Brain immune cells—for example, macrophages and dendritic cells—present in the meninges and choroid plexus. These parenchymal macrophages are called microglial cells. Comparatively, these are more dormant than other tissue macrophages; however, they can only react to inflammatory stimuli by secreting prostaglandins and pro-inflammatory cytokines. Microglia regulates cytokines and the inflammatory process in the brain and is involved in major depression [39]. TNF-α is a leading pro-inflammatory cytokine inhibiting neurogenesis and has a negative effect on hippocampal adult neurogenesis, whereas anti-TNF treatment promotes neurogenesis [40]. Additionally, both neural and non-neuronal brain cells can express their receptors for these mediators [12].

In the brain, TNFR1 seems to show a constitutive pattern of expression, whereas TNFR2 is generally expressed under stimulatory conditions [41]. The maximum concentrations of TNF-α receptors are found in several regions of the brain such as the hypothalamus, hippocampus, amygdala, and prefrontal cortex, which plays an important role in the regulation of emotion and is supposed to be involved in depression [42]. Astrocytes and microglia cells of the CNS secrete TNF-α in reaction to inflammatory or infectious stimuli [43]. Meanwhile, most brain neurons, in particular stimulatory conditions, also have the ability to release cytokines [44].

### 2.3. Brain Signaling by Peripherally Produced TNF-α

Tissue injury and infection can stimulate the production of TNF-α in periphery. TNF-α is a large molecule and circulatory cytokines naturally cannot cross the blood-brain barrier (BBB) in normal physiological situations. However, it reaches the brain by using several immune-mediated pathways and transfers the signals from the periphery to CNS [45,46]. The fast transmission pathway is one of them, in which primary afferent nerves innervate the site of inflammation. Another pathway called slow transmission pathway consists of cytokines initiating from the circumventricular organs and choroid plexus, diffusing into the brain parenchyma by bulk transmission [47]. In the third pathway, TNF-α is transported across the BBB under specific saturable transport systems.

## 3. Evidence from Animal and Human Studies

### 3.1. Animal Models

Much research has collected substantial evidence from animal studies that support the role of TNF-α in depression [48,49,50,51]. A study reported in 2012, in which researchers administered TNF-α in animal models, which induced many symptoms such as: decreased social behavior, locomotors activity and anhedonia, suppression of food intake, sleep abnormalities, fatigue, and alterations in cognition. Additionally, these symptoms were blocked by co-administration of an anti-TNF-α antibody [25]. These symptoms are collectively known as “sickness behavior”, which was similar to human depression patients. Interestingly, the depressive-like phenotype has been successfully diminished or eliminated with the administration of conventional or novel antidepressants (m-trifluoromethyl-diphenyl diselenide, agmatine and α-tocopherol) in animal models such as mice and rats. Additionally, the use of selective serotonin reuptake inhibitors (SSRI) and tricyclic antidepressants have been shown to decrease immune activation and the levels of TNF-α induced olfactory bulbectomy and chronic mild stress in rats [52,53]. Moreover, Simen *et al.* proved that the removal of TNFR1 and TNFR2 exhibited an antidepressant-like behavior in the tail suspension test (TST) and forced swimming test (FST) as compared with the wild type mice [54].

### 3.2. Clinical Studies

A recent meta-analysis calculating cytokine concentrations in MDD patients has found significantly higher concentrations of TNF-α in depressed subjects as compared with control subjects. Another study conducted in Europe recruited a psychiatric patient population, which had shown high levels of TNF-α and soluble TNF-receptors (p55 and p75) in past history patients or those currently facing depression [55]. Another report also illustrated that TNF-α levels were significantly higher in the plasma of suicide attempters [56] and in the postmortem brains of suicide victims as compared to non-suicidal depressed patients and healthy controls [57]. In addition, clinical studies suggest that TNF-α can induce “sickness behavior” in viral or bacterial infection patients [58]. At the preclinical level, *in vitro* studies with human whole blood, cultured lymphocytes and monocytes and studies with rat brain slices have reported that several classes of antidepressants are able to inhibit the production of pro-inflammatory cytokines including TNF-α [59,60].

Altogether, these studies indicated that TNF-α may be capable of causing mood swings and depression, and central administration of it would be an innovative method to investigate the inflammatory component of depressive disorder.

## 4. The Pathophysiologic Role of TNF-α in Depression

**Figure 1:**
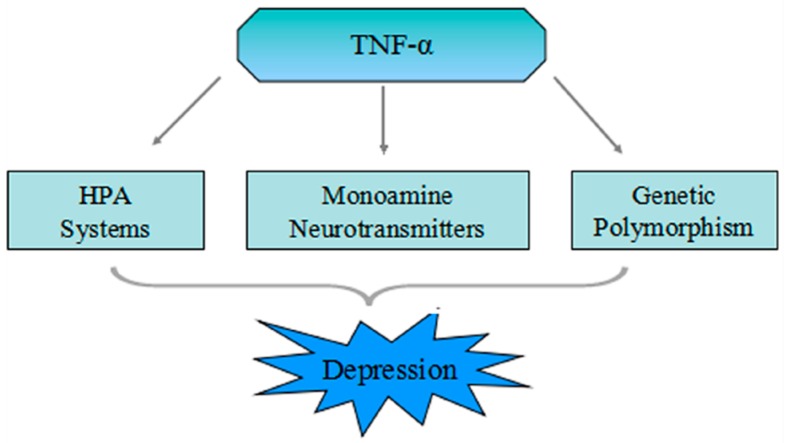
Scheme of the three different mechanisms which show correlation between tumor necrosis factor (TNF)-α and major depressive disorder (MDD). Peripheral TNF-α stimulated by infection and tissue damage cross the blood-brain barrier (BBB) through fast transmission pathway involving primary afferent nerves a slow transmission pathway or saturable transport system. Furthermore, single nucleotide polymorphisms in the promoter region of the *TNF-α* gene can induce high binding affinity of nuclear factors to the TNF promoter, which can elevate the level of transcription activity and secretion of TNF-α. TNF-α may cause depression or depressive symptoms through HPA-axis activation, neuronal serotonin transporter activation, and the motivation of the indoleamine 2,3-dioxygenase, which leads to tryptophan depletion.

Many observation have shown that effects of the cytokine system, in which TNF-α is a part, on serotonin metabolism as well as on the hypothalamic-pituitary-adrenal (HPA)-axis, may induce changes in the structure and function of the brain, possibly leading towards the development of depression [61]. There are three leading mechanisms which might relate the TNF-α system to the pathophysiology of depression (Figure 1).

### 4.1. Mutual Influence of the TNF-α and HPA System

The HPA-axis is the main neuroendocrine system that controls stress related physiological response, and, as a result, drives how an organism might adapt its own behavior or environment in order to accommodate that stress [62]. In a short summary of the HPA-axis circuit, the awareness about stress starts a signal in the paraventricular nucleus (PVN) of the hypothalamus. There are neurons in the PVN which produce and release corticotrophin-releasing hormone (CRH), which is moved through the hypophyseal portal system and attaches to the particular receptor in the anterior pituitary (adenohypophysis), initating the production and secretion of adrenocorticotropic hormone (ACTH) from the anterior pituitary and secreted it into the circulatory system. Finally, it controls the production and release of glucocorticoids from the adrenal cortex [63]. The normal function of the HPA-axis might be altered with the usual aging process; however, its activity was enhanced in stressful or traumatic conditions, immunosuppression, as well as changes in noradrenergic, dopaminergic, and serotonergic pathways [64]. Chronic activation of the HPA-axis is associated with glucocorticoid resistance, and this has been reported in almost 50% of cases with mood disorders [65].

The stimulation of the cytokine system might be a possible cause of depression-related activation of the HPA-axis [55,66]. The stress reaction system is associated in a complex manner with pro-inflammatory signaling. It has been already reported that the release of TNF-α elevates the levels of ACTH, CRH, and glucocorticoids (GC), which has a direct effect on pituitary gland and hypothalamic cells [67,68], and upregulates the HPA-axis [69]. Primarily, this system’s hyperactivity has endorsed glucocorticoid receptor (GR) resistance, while, secondarily, it may also be due to low expression of GR or a fault in normal function of GR [61]. The GR, as one of GC’s signal receptors, is expressed in the hypothalamus, prefrontal cortex, and hippocampus [70]. It acts as a ligand-activated transcription factor upon activation to regulate metabolism for fight-or-flight responses as well as stopping more GC production by suppressing HPA-axis activation [71,72]. Impaired GR cannot effectively suppress HPA-axis activity, which, in turn, leads to more hyperactivity, creating a vicious cycle.

Cytokine signaling molecules such as c-Jun N-terminal kinases (JNK), NF-κB, and signal transducers and activators of transcription-5 (STAT5) have been shown to control GR. Considerable evidence demonstrates that TNF-α encourages glucocorticoid resistance [73,74,75,76]. The functionality is controlled by inhibiting the entry of the cortisol-GR receptor complex into the nucleus (by inducing JNK) and also by inhibiting the binding of the complex to the DNA (by inducing NF-κB), which may lead to changed expression of GR in cells [77]. Variations of GC signaling play a leading role in the development of depression [78]. These could be caused by subtle fluctuations in GR function as a result of functional polymorphisms [79]. To restore the control of the HPA-axis and increase GR, antidepressants have been the best alternative.

In contrast, findings from Schuld *et al.* have suggested that the hyperactivity of chronic HPA system in depressed patients inhibited the production of inflammatory cytokines [80]. Another study reported the effect of both the TNF-α system and the HPA system in depressed patients without inflammatory diseases, and the level of TNF-α was inversely related with the ACTH response to the collective dex/CRH test; thus, it has been concluded that the hyperactivity of HPA-axis in acute depression patients downregulate the TNF-α system activity, while after remission, as HPA-axis activity become normal, the TNF-α system appears to obtain its influence on the HPA system [81].

### 4.2. TNF-α Increases Activation of the Neurotransmitter Transporter

The pro-inflammatory cytokines depressogenic effects are mostly due to their action on the neurotransmitter serotonin (5-HT) homeostasis, and its deficiency is recognized as a key pathophysiologic feature of depressive disorders and the target of most antidepressants [82]. Several drugs, such as selective serotonin reuptake inhibitors (SSRI), are used for depression treatment, because it deactivates the serotonin transporters (SERT). Certainly, Zhu *et al.* concluded that TNF-α motivated the 5-HT uptake in mouse midbrain and striatal synaptosomes and rat embryonic raphe cell line [83]. However, knockout of TNF-α receptors in mice increased the levels of 5-HT in the synaptic cleft [84]. Moreover, a variety of studies have reported that TNF-α could reduce 5-HT availability by activating SERT via trafficking-dependent and independent processes through p38 MAPK activation [48,50,51,85]. These observation suggested that TNF-α can intensely regulate the the activety of neuronal serotonin transporter.

Recently, it has been demonstrated that TNF-α raised the brain monoamine metabolism, which caused anhedonia in mice. TNF-α has elevated the dopamine metabolite homovanillic acid without disturbing dopamine levels itself [86]. On the basis of above-mentioned observation, TNF-α not only enhanced serotonin transporter activity, but also the activity of dopamine transporter in the brain, resulting in depression symptoms.

### 4.3. TNF-α Stimulates the Indoleamine 2,3-Dioxygenase (IDO)

Emerging research suggests that disturbances in tryptophan metabolism may be associated with pro-inflammatory cytokines and depression [87,88,89]. There are two main metabolic pathways for tryptophan: (1) the kynurenine (KYN) pathway; (2) the serotonin-synthesis pathway. A change in the normal level of tryptophan metabolism from the serotonin towards the KYN pathway may be involved in the pathophysiology of depression [90]. Indoleamine 2,3-dioxygenase (IDO) is mostly expressed intracellularly as a inducible forms or constitutive form in tissue macrophages and blood monocytes, including microglial cells within the brain parenchyma [91]. Owing to IDO altering tryptophan along the kynurenine pathway, any variations in its enzymatic activity can change the brain tryptophan metabolism [92]. The elevated use of serotonin and its precursor tryptophan due to IDO activation may explain the low availability of serotonin in depression.

It has been reported that TNF-α directly or indirectly affects the tryptophan metabolism by stimulating IDO [93,94,95,96,97]. On one side, activated IDO attenuates the brain conversion of tryptophan into serotonin, while, on the other side, it initiates the KYN pathway, all of which leads to the depletion of serotonin. Decline of serotonin level is generally related to depression; however, depression resulting from TNF-α-mediated IDO activation and KYN production has additional serotonin-independent effects. For instance, depressive behavior in mice has been associated with administering KYN alone [98]. Moreover, KYN is further metabolized to several catabolisms of tryptophan, including kynurenic acid and the neurotoxic substances quinolinic acid (QUIN) and 3-hydroxykynurenine [99]. Both neurotoxic end products promote glutamate release through activation of *N*-methyl-d-aspartate (NMDA) receptors as well as producing neurotoxicity, resulting in glutamate excitotoxic cell death [100]. In contrast, kynurenic acid acts as an antagonist of NMDA receptors and can be considered neuroprotective [101]. Another important point to note is that activation of IDO additionally leads to the production of glutamatergic agonists [102]. The role of increased glutamatergic neurotransmission in the pathogenesis of depression is inconclusive [103].

Therefore, we can hypothesize three mechanisms of how TNF-α may lead to depression or depressive symptoms: (1) modification of the HPA-axis; (2) activation of neurotransmitter transporter; (3) stimulation of IDO, which leads to tryptophan depletion and initiates the KYN pathway.

## 5. The Genetic Polymorphism of TNF-α in MDD

Functional polymorphisms in the promoter region of regulatory genes predict phenotypes of interest in interaction with predisposing biological factors or behavioral [104,105]. Since dysregulation of inflammatory cytokines play a crucial role in the mechanisms underlying depression, and polymorphisms in cytokine genes are associated with increased secretion or expression of inflammatory biomarkers, there is considerable evidence supporting the relationship between the risk of depressive disorder and single nucleotide polymorphisms (SNPs) in inflammation-related genes [106,107,108].

### 5.1. The Genetic Polymorphism of TNF-α

The human *TNF-*α gene is positioned in the class III region of the major histocompatibility complex (MHC), on the small arm of chromosome 6 (6p21.1–21.3) [109]. It spans about three kilo-bases and contains four exons, encoding a protein consisting of a signal peptide composed of 76 amino acid residues and the 157 amino acid residues of the mature peptide. Multiple genetic polymorphism loci are present in the *TNF-α* gene region. According to the National Center for Biotechnology Information (NCBI), to date, more than 391 SNPs have been identified (Figure S1). Among these SNPs, the promoter region of the *TNF-α* gene at nucleotide -308 (also referred to as rs1800629), with G to A substitution polymorphism, was the most extensively studied. There are two alleles at the polymorphic site, TNF-α -308G and TNF-α -308A. In normal populations, TNF-α-308G homozygosity is the predominant genotype [110]. The A allele of this polymorphism can lead to high binding affinity of nuclear factors to the TNF promoter, resulting in a high level of transcription activity and secretion levels of TNF-α [111,112,113]. Thus, TNF-α rs1800629 polymorphism was proposed to have a significant functional effect.

### 5.2. Association of TNF-α Genetic Polymorphism in Major Depressive Disorder (MDD)

Up to now, the role of *TNF-α* gene polymorphisms in susceptibility to MDD has been extensively investigated in different ethnicities, especially rs1800629, but conflicting results were obtained due to the heterogeneity of the genetic background among populations, the number of cases in these studies, and the complexity of the pathogenesis of depression (Table 1). Jun *et al.* conducted for the first time the case-control association study regarding *TNF-α* rs1800629 polymorphism between patients with MDD and the controls. They demonstrated that the ‘‘high producer’’ A allele and the AA genotype of the rs1800629 increase depression susceptibility in the Korean population. Then, in another study, rs1800629 A allele was also found to be associated with post-stroke depression. However, conflicting results were reported by Clerici *et al.* [114] who observed a different allele distribution of rs1800629 among a sample of 84 Italian outpatients affected by bipolar disorder type I, bipolar disorder type II, or MDD. In particular, the percentage of A carrying subjects was lower in patients with MDD. Another study, looking at individuals with late-life MDD in elderly people without dementia, suggested that the presence of the GG genotype significantly increased the risk of developing MDD. Recently, Kim *et al*. confirmed that the rs1800629 GG genotype was an independent risk factor for suicide attempts in MDD [115]. These findings supported the existence of a genetic profile related to TNF-α in patients affected by depression. 

However, in patients with a single depressive episode with or without stressful life events prior to MDD, no involvement was found for the TNF-α SNPs rs1800629 and rs361525, or two others in the promoter region at positions -1031(T/C) and -857(C/T). This finding is in line with reports from Misener and colleagues [117], who found no significant association with childhood depression and these same TNF-α polymorphisms. A further polymorphism at position -850(C/T) was investigated for the first time in post-stroke depression patients, with negative findings. We identified one further study concerning the TNF-α A-308G polymorphism (rs1800629) in relation to IFN-α induced depression. The authors found a significant association with labile anger and fatigue but not with depression.

Bosker *et al*. [120] used data from the Genetic Association Information Network (GAIN) genome-wide association study (GWAS) in MDD to explore previously reported candidate genes and SNP associations in MDD. In a sample of 1738 MDD patients, 57 genes were identified and 92 SNPs mapped. The TNF-α rs769178 was the only gene found to be related to depression and that remained significant after correcting for multiple testing.

## 6. The Therapeutic Implications of TNF-α in MDD

### 6.1. The Potential Benefits of TNF-α Antagonists on Depression

Excess levels of TNF-α may play a pivotal role in the pathophysiology of depression. Consequently, several TNF-α antagonists have been developed to block the action of TNF-α in depressive patients [124]. Indeed, inhibition of TNF-α may be achieved with a monoclonal antibody such as infliximab [125], adalimumab or golimumab, with a circulating TNF-α receptor fusion protein such as etanercept [126], or with certolizumab, a PEGylated Fab fragment of a humanized TNF-α monoclonal antibody. The antidepressant effect of these drugs has already been successfully reported in animal models and depression patients with chronic inflammatory disorders.

Using chronic unpredictable mild stress combined with separation, Karson *et al.* reported that chronic administration of infliximab decreased chronic unpredictable mild stress (CUMS)-induced depression-like behaviors back to or lower than presumably the normal levels observed in saline-control rats [127]. In another study, Bayramgürler *et al*. explored whether long-term etanercept treatment could affect the anxiety- and depression-like neurobehaviors in rats without a chronic inflammatory or stressful condition by various behavioral methods. The anxiety- and depressive- like neurobehaviors of the animals as assessed with an elevated plus maze and forced swimming tests, respectively, were found significantly decreased after the etanercept treatment. However, etanercept did not alter total locomotors activity levels, which indicated that differences observed in emotional tests were not mediated by the psychomotor activation of etanercept. These findings suggested that TNF-α has a role in the modulation of emotional processes, and its inhibition may represent a novel strategy for the treatment of affective disorders [128]. Meanwhile, Krügel *et al.* demonstrated treatment with etanercept significantly that reduced the restraint-induced depression-like effects, resulting in reduced immobile time in the FST and intensified climbing behavior, both similar to the antidepressive-like effect of imipramine. They speculated that antidepressant effects of etanercept may be caused by enhancement of serotonergic or noradrenergic neurotransmission or normalization of stress hormone secretion [129]. Very recently, Şahin *et al.* has showed that chronic infliximab treatment prevented the CUMS-induced cognitive impairments as well as the reduction in the levels of hippocampal brain-derived neurotrophic factor (BDNF). These results suggest that infliximab improves the spatial and emotional memory impairments induced by chronic stress in rats, likely through its effects on hippocampal function by modulating inflammation [130].

It is well known that TNF-α blockers are very effective in the treatment of chronic inflammatory disorders like psoriasis, Crohn’s disease (CD), and ankylosing spondylitis (AS), which can co-exist with depression. However, recent clinical trials have shown that TNF-α blockers were also effective in decreasing depressive symptoms associated with these disorders. In one of these trials, Tyring and co-workers firstly demonstrated that, compared to placebo administration, etanercept treatment led to an improvement of at least 50% in depression rating scales at week 12. However, the improvement in symptoms of depression was not strongly correlated with the improvement in psoriasis area and severity index (PASI) in this study [28]. Subsequently, Bassukas and co-workers reported that treatment with infliximab resulted in stabilization or improvement of the manifestations of psychiatric morbidity in three psoriasis patients with overt psychiatric disorders, including recurrent depression and bipolar disorder [131]. Finally, Menter *et al.* showed that adalimumab treatment reduced symptoms of depression and improved health-related quality of life (QOL) in addition to improving psoriasis [132]. In contrast to Tyring *et al*. [28] they reported that reductions in depression symptoms were correlated with PASI. As a matter of fact, Lichtenstein *et al.* [133] have found that infliximab significantly improved QOL in patients with active CD, increasing their ability to work and participate in leisure activities and decreasing fatigue, depression and anger [133]. Then, Persoons *et al.* also reported the beneficial effects of infliximab on depression and psychological well-being in active CD [134]. Likewise, Minderhoud *et al.* have demonstrated that the administration of infliximab in CD significantly reduced depression scores and improved the QOL as well as fatigue in a four-week follow-up study, although a clear role of cytokines could not be substantiated [135]. Up to now, there are only two studies about the effect of TNF-α blockers in AS patients, and both of them suggested that treatment with TNF-α antagonism caused a significant decrease in depression-anxiety scores and disease activity and a significant increase in QOL, and this change in disease activity was not correlated with changes in depression and anxiety scores [27,136]. These observations together with the theoretical background reported in this article leads to the hypothesis that TNF-α blockers may reduce the effect of pro-inflammatory cytokines and reverse depressive symptoms associated with chronic inflammatory disorders both of which are thought to be associated with increased levels of TNF-α.

In view of the association between inflammatory cytokines and treatment resistance [137,138], it is interesting to verify whether inhibiting inflammatory cytokines might have therapeutic potential in treatment resistant depression (TRD). Raison *et al.* for the first time performed a randomized double-blind, placebo-controlled clinical trial of infliximab *versus* placebo for antidepressant non-responders with major depression. Surprisingly, no differences in clinical response were found between infliximab and placebo in the group as a whole. However, there was a significant interaction between baseline inflammation and treatment response, with patients exhibiting high initial markers of inflammation performing clinically better [139]. Mehta *et al.* further examined additional predictors and targets of response to infliximab in patients with TRD, they found baseline transcriptional signatures reflective of alterations in glucose and lipid metabolism predicted antidepressant response to infliximab, and infliximab response involved regulation of metabolic genes and inhibition of genes related to innate immune activation [140]. Studies by Stellwagen and Malenka indicate that synaptic scaling in response to prolonged blockade of activity is mediated by the pro-inflammatory cytokine TNF-α and glia are the source of the TNF-α that is required for this form of synaptic scaling [141,142]. TNF-α is integral for homeostatic synaptic plasticity, which could contribute to the negative effects of TNF-α antagonists in patients that do not get elevated inflammatory markers, as reported by Raison *et al*. [139] Moreover, results from Weinberger *et al.* [134] suggested that administration of infliximab in patients with TRD and high inflammation could improve sleep continuity independently of depressive symptoms.

### 6.2. The Effect of Antidepressant Medication Treatment on the Level of TNF-α

The research on biomarkers that predict antidepressant response has been recognized as remarkably difficult, and the literature is spoiled with conflicting observations regarding an array of putative genetic and physiological predictor variables. Given that TNF-α has been recognized to be involved in the pathogenesis of MDD, it is worthy to examine whether TNF-α is associated with antidepressant medication treatments and could serve as putative biomarkers for clinical response. Multiple studies have evaluated the effects of antidepressants on TNF-α, and the results are not consistent. Owing to heterogeneity and the complex nature of MDD, TNF-α level was increased [143], decreased [144] or unchanged [26] after pharmacological treatments. Even among treatments with the same antidepressants, the change of TNF-α level in MDD patients is also controversial. For example, Kraus’s group in 2002 found that the plasma TNF-α level was unchanged following venlafaxine, the most common antidepressant, therapy in depressed patients. Their observation was based on a study recruiting 11 MDD patients treated with mirtazapine and nine patients treated with venlafaxine for four weeks [145]. Piletz *et al.* also reported that MDD patients had higher plasma TNF-α levels compared to healthy subjects, and this value further increased after eight-week venlafaxine treatment [146]; In a follow-up study, total 61 MDD patients received eight-week venlafaxine treatment, and they were divided into responders and non-responders according to the reduction rate of HRSD-17. Prior to the treatment of venlafaxine, both responders and non-responders showed similarly higher levels of plasma TNF-α, which significantly decreased following eight-week treatment of venlafaxine. Compared with the non-responder group, the responder group had a greater reduction in TNF-α, which positively correlated with the reduction rate of HRSD-17 [147].

Faced with controversial clinical findings, Strawbridge *et al*. recently conducted a meta-analyses examining data from 35 studies (TNF-α in 11 studies) that investigated pro-inflammatory cytokines before and after treatment in depressed patients together with a measure of clinical response [148]. TNF-α level was not significantly changed when simply looking at the effects of treatment. However, there was a differential effect when treatment response was taken into account: levels of TNF-α significantly decreased in treatment responders but not in non-responders. This implies that maintenance of heightened levels of inflammation may at least contribute to treatment refractoriness, and thus that anti-inflammatory agents might provide a mechanism for treatment resistance in individuals with persistent high levels of TNF-α.

There are several ways that TNF-α could contribute to the action of antidepressants [149]. First, anti-TNF-α may increase the efficacy of the drugs or otherwise be permissive for drug action. Anti-TNF-α does increase SERT expression in astrocytes [150] and stimulate uptake [83], so it could influence the efficacy of fluoxetine in that manner. It is unclear if the norepinephrine transporter is similarly regulated; Second, anti-TNF-α can increase the synthesis of brain-derived neurotrophic factor (BNDF), thought to be important for the response to antidepressants [151]; Third, anti-TNF-α is also known to increase neurotransmitter receptor surface expression, particularly of α-amino-3-hydroxy-5-methyl-4-isoxazole-propionic acid receptor (AMPAR). Changes in glutamate signaling are thought to be important in depression and the response to antidepressants [152]. Fluoxetine increases AMPAR expression and synaptic content in the cortex and hippocampus [153]. Anti-TNF-α may also exert effects through the regulation of other neurotransmitter receptors. For example, the 5-HT1A auto-receptor can also set responsiveness to antidepressants [154], but its regulation by TNF-α has not been tested.

## 7. TNF-α and MDD with Autoimmune Diseases

Patients with autoimmune disease more frequently suffer from major depressive episodes. This fact might be associated with the physical disability and multiple stresses existing with chronic disease conditions. Currently, studies designed with the objective to explore the association between depression and pro-inflammatory cytokines in patients suffering with depression and autoimmune diseases, such as systemic lupus erythematous (SLE) and multiple sclerosis (MS), are not common. A recent data found that serum TNF-α levels were significantly higher in SLE patients with depression. Additionally, it was also independently associated with more severe depression symptoms in these patients. These finding propose a potential impact of TNF-α on depression symptoms in SLE patients [155]. Similarly, Postal *et al.* observed increased levels of TNF-α in childhood-onset SLE patients with moderate/severe depression as compared to patients with no/mild depression [156]. There is only one study reported on MS patients with depression. The study investigated the connection between depressive symptoms, neurological disability and cytokine mRNA expression levels of Th1-type and Th2-type cytokines in early diagnosed MS patients. Their data support a positive correlation of TNF-α mRNA expression levels and depressed symptom during an acute attack in MS patients [157]. The relationship between TNF-α and depression in patients with autoimmune disease may be explained by two main theories. One hypothesis is that chronic stress that originated from long-term use of corticosteroids undermines corticosteroid-receptor signaling [73]. Another possible interpretation is the inflammation hypothesis of depression [42]. Pro-inflammatory cytokine may act on neurotransmitter metabolism, neuroendocrine function, neurogenesis and synaptic plasticity related to depression. Patients with autoimmune conditions have higher levels of pro-inflammatory cytokine [158]. Consequently, they have a more expressive interaction.

## 8. Conclusions

As mentioned above, both direct and indirect evidence recommends that TNF-α might contribute to the etio-pathogenesis of MDD and the mechanisms of antidepressant treatment. Peripheral TNF-α motivated via infection and tissue damage can cross the BBB through fast transmission pathway involving primary afferent nerves, a saturable transport system, or a slow transmission pathway. The secretion of TNF-α has been revealed to elevate the levels of ACTH, cortisol and CRH, which have a direct effect on the HPA-axis. The upregulation of the HPA-axis can cause depression. Furthermore, TNF-α also activates neurotransmitter transporters and indirectly consume serotonin and its precursor tryptophan, which leads to decreased monoamine neurotransmitters in synaptic cleft.

Interestingly, anti-TNF-α antagonists have already been revealed to have antidepressant effects or improve antidepressant response. Moreover, high levels of TNF-α and other inflammatory markers forecast non-responsiveness of patients to standard antidepressants. Therefore, normalization of peripheral TNF-α may be regarded as alternative treatment or preventative strategies, and potential biomarkers for treatment response. Upcoming research needs to advance the existing discoveries and initiate the teasing out of some of the unknown issues highlighted such as—can TNF-α inhibitor therapy recover certain subgroups of populations with depression who present with higher inflammatory biomarkers, and may existing levels of TNF-α or other inflammatory biomarkers in individuals with depression indicate who is likely to respond to therapy? Moreover, maybe there are some other characteristics (biological, psychological and social) that recognize who are most likely to respond to TNF-α inhibitor therapy? Some antidepressant effects are observed by presumably blocking peripheral TNF-α, for example with TNF-α antibodies and neutralizing agents that are administered peripherally and do not cross the blood brain barrier. What are the proposed mechanisms underlying the actions of these agents on brain behavior and function? Whether TNF-α gene polymorphism’s association of its blood levels and treatment in MDD patients? In the experiment to figure out the mechanism, there is still uncertainty about whether TNF-α inhibitors have any direct effect on depression or whether they are indirectly improving depression by recovering the underlying physical condition.

In conclusion, recognizing the influence of TNF-α on neurotransmitter systems and obtaining a better understanding of the consequences of altered inflammatory responses may support not only MDD but also other neuropsychiatric illnesses.

## Figures and Tables

**Table 1:** Studies examining the relative contribution of tumor necrosis factor (TNF)-α gene polymorphisms on depression.

Study (Year)	Type of Study	SNP	Ethnicity	Sample (Case/Control)	Genotyping	Diagnostic Criteria	Main Conclusion
Jun *et al.* [116]	Case-control	-308G/A	Korea	108/125	PCR-RFLP	DSM-IV	*TNF-α* -308 A allele affected MDD susceptibility
Misener *et al*. [117]	Family-based association of COMD	-238A/G -308G/A -857C/T -1031T/C	Caucasian Roma African	460 children from 384 families	TaqMan^®^ 5′ nuclease Assay	DSM-IV ISCA-D	No evidence supported *TNF-α* as genetic risk factors contributing to COMD
Cerri *et al.* [118]	Case-control	-308G/A	Italy	50/240	PCR-SSP	DSM-IV GDS ≥ 15 MMSE ≥ 24	*TNF-α* -308 G/G genotype related with a greater risk of developing MDD
Clerici *et al.* [114]	Case-control	-308G/A	Italy	32 MDD 32BD I 20BD II/363	PCR-SSP	DSM-IV Physical examination	No association of *TNF-α* polymorphisms with MDD
Lotrich *et al.* [119]	Prospective study	-308G/A	NR	105 IFN-α induced depression cases in HCV patients	TaqMan^®^ 5′ nuclease Assay	DSM-IV BDI-II AIAQ TNF-α levels	TNF-α -308 A allele associated with labile anger and fatigue but not with MDD
Bosker *et al.* [120]	GWAS	55 gene 92 SNP	Multi-ethnic	1738/1802	NR	GAIN	*TNF-α* was identified as the only gene associated with MDD (rs76917)
Kim *et al.* [121]	Case-control	-238A/G -308G/A	Korea	29 major post-stroke depression cases/199	PCR-SSP	DSM-IV MINI	Higher frequencies of *TNF-α*-308 A in major post stroke depression
Haastrup *et al*. [122]	Case-control	-238A/G -308G/A -857C/T -1031T/C	Denmark	288/335	Qiagen^®^ FlexiGene kit	SCAN IRLE	None of the examined *TNF-α* alleles were differently distributed among MDD
Holtzman *et al*. [123]	Cross-sectional study	-308G/A	Canada	93 MDD cases with end stage renal disease	Sequenom iPLEX assay	BDI-II	No associations with depression were found
Kim *et al*. [115]	Case-control	-308G/A	Korea	204 MDD cases with attempted suicide/97	PCR-SSP	DSM-IV	*TNF-α*-308G/A polymorphism was an independent risk factor for suicide attempts in MDD

Abbreviation: PCR-RFLP = Polymerase Chain Reaction-Restriction Fragment Length Polymorphism; PCR-SSP = Polymerase Chain Reaction-Sequence Specific Primers; DSM-IV = The Diagnostic and Statistical Manual of Mental Disorders; GDS = Geriatric Depression Scale; MMSE = Mini-Mental State Evaluation; BDI-II = Beck Depression rating scale; AIAQ = Anger Irritability and Assault Questionnaire; SCAN = Clinical Assessment in Neuropsychiatry; COMD = Childhood-Onset Mood Disorders; MINI = Mini International Neuropsychiatric Interview; IRLE = Interview for Recent Life Events; GAIN = Genetic Association Information Network; GWAS = Genome-Wide Association Study. A suicide attempt was defined as self-harm with at least some intent to end one’s life; NR = None Report.

## Supplementary Materials

Supplementary materials can be found at http://www.mdpi.com/1422-0067/17/5/733/s1.
