# Supplementary material for: Pathogenetic and Therapeutic Applications of Tumor Necrosis Factor-α (TNF-α) in Major Depressive Disorder: A Systematic Review
Source: Int J Mol Sci. 2016 May 14;17(5):733. doi: 10.3390/ijms17050733 (PMC4881555; doi:10.3390/ijms17050733)
Supplement: Supplementary file 1 [file ijms-17-00733-s001.pdf]

Int. J. Mol. Sci. **2016**, *17*, 733; doi:10.3390/ijms17050733

Supplementary Materials: Pathogenetic and Therapeutic Applications of Tumor Necrosis Factor-α (TNF-α) in Major Depressive Disorder: A Systematic Review

Ke Ma, Hongxiu Zhang and Zulqarnain Baloch

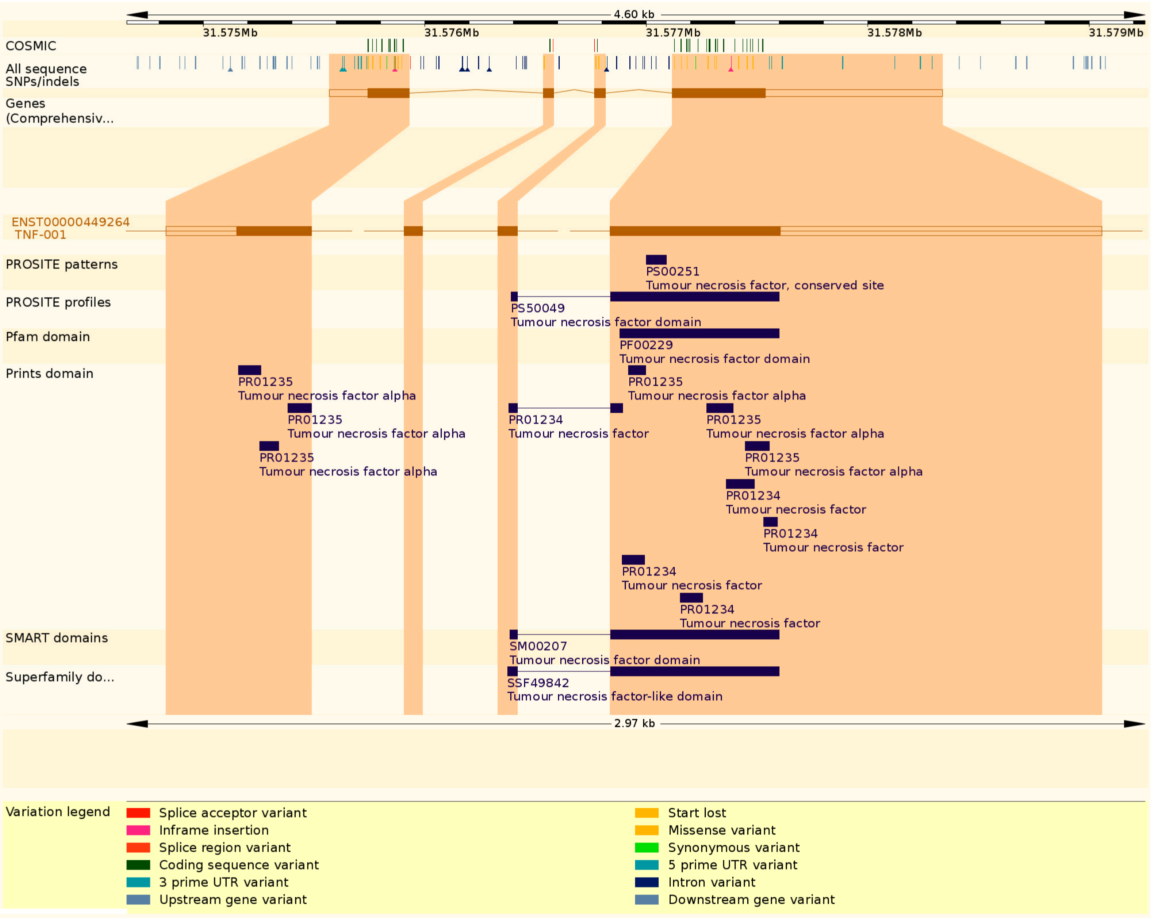

Figure S1. The genetic polymorphism of TNF-α. The human *TNF-α* gene is positioned in the class III region of the major histocompatibility complex (MHC), on the small arm of chromosome 6 (6p21.1–21.3). It spans about 3 kilobases and contains 4 exons, encoding a protein consisting of a signal peptide composed of 76 amino acid residues, and the 157 amino acid residues of the mature peptide. Multiple genetic polymorphism loci are present in the *TNF-α* gene region. According to date from national center for biotechnology information (NCBI), more than 391 SNPs have been identified.
